# Effects of BD1047, a σ_1_ receptor antagonist, on the expression of mTOR, Camk2γ and GSK-3β in fluvoxamine-treated N2a cells

**DOI:** 10.3892/etm.2013.1438

**Published:** 2013-12-05

**Authors:** DONG-AN SU, RI-YUE JIANG, NING LIU, LIANG-CAI DING, DA-MING WANG, HAI-YING YU, EN-SHI YAN, MEI-HUA ZHU, BIN ZHU

**Affiliations:** 1 Department of Anesthesiology, The PLA 102^nd^ Hospital, Second Military Medical University, Changzhou, Jiangsu 213003, P.R. China; 2 Department of Radiation Oncology, The Third Affiliated Hospital of Soochow University, Changzhou, Jiangsu 213003, P.R. China; 3 Department of Critical Care Medicine, The Third Affiliated Hospital of Soochow University, Changzhou, Jiangsu 213003, P.R. China; 4 Department of Psychiatry, The PLA 102^nd^ Hospital, Second Military Medical University, Changzhou, Jiangsu 213003, P.R. China; 5 Department of Anesthesiology, Brain Hospital Affiliated to Nanjing Medical University, Nanjing, Jiangsu 210029, P.R. China

**Keywords:** σ_1_ receptor antagonist, fluvoxamine, mammalian target of rapamycin, Ca^2+^/calmodulin-dependent protein kinase 2γ, glycogen synthase kinase-3β

## Abstract

Fluvoxamine, a common antidepressant agent, is designed to exert its pharmacological effect by inhibiting synaptic serotonin reuptake. However, increasing evidence has demonstrated that σ_1_ receptors are likely to be involved in the mechanism of action of fluvoxamine. The present study aimed to observe the effects of fluvoxamine on the expression levels of mammalian target of rapamycin (mTOR), Ca^2+^/calmodulin-dependent protein kinase 2γ (Camk2γ) and glycogen synthase kinase-3β (GSK-3β) in fluvoxamine-treated N2a cells and attempted to elucidate whether σ_1_ receptors mediate the pharmacological effects of fluvoxamine. The N2a cells were randomly divided into three groups (each n=6): DMEM group (D group), 0.5 μmol/l fluvoxamine group (F group) and 0.2 μmol/l BD1047 (a σ_1_ receptor antagonist) + 0.5 μmol/l fluvoxamine group (BF group). Western blotting was used to determine the expression levels of mTOR, Camk2γ and GSK-3β in the cultured N2a cells after two days of incubation. The F group exhibited significant increases in the expression levels of mTOR and Camk2γ and a significant reduction in the expression levels of GSK-3β compared with those in the D group (P<0.01). By contrast, the BF group demonstrated significant reductions in the expression levels of mTOR and Camk2γ and a significant increase in the expression levels of GSK-3β, compared with those in the F group (P<0.01). These results suggest that σ_1_ receptors mediate fluvoxamine-elicited changes in the expression levels of mTOR, Camk2γ and GSK-3β in N2a cells, which indicates that σ_1_ receptors are likely to be involved in the pharmacological effects of fluvoxamine.

## Introduction

Fluvoxamine, a common and widely used antidepressant agent, is intended to exert its therapeutic effects in patients with depression by inhibiting the reuptake of serotonin in synaptic clefts (1). Certain studies have supported the theory that fluvoxamine exerts therapeutic effects not only on depression but also on schizophrenia and bipolar depression (2,3). Consequently, the conventional serotonin hypothesis is not able to not fully elucidate the pharmacological mechanisms of action of fluvoxamine.

σ receptors are recognized as non-opioid, intracellular receptors that modulate a variety of types of signal transduction in cells (4). A number of studies have demonstrated the existence of at least two subtypes of the σ receptor, σ_1_ and σ_2_, and σ_1_ receptors are expressed in numerous organs such as the brain, liver, pancreas, spleen and adrenal glands (5–7). The wide distribution of σ_1_ receptors in a variety of tissues suggests a critical role in living systems (6). A previous study by Niitsu *et al*(8) suggested that a σ_1_ receptor agonist caused a significant therapeutic effect in the treatment of schizophrenia. Additionally, SA4503, a σ_1_ receptor agonist, alleviated schizophrenia symptoms in an animal model (9). A study using fluvoxamine for the treatment of a patient with schizophrenia suggested that σ_1_ receptors are probably associated with the mechanism of action of fluvoxamine (10).

Mammalian target of rapamycin (mTOR), Ca^2+^/calmodulin-dependent protein kinase 2γ (Camk2γ) and glycogen synthase kinase-3β (GSK-3β) are three fundamental biomarkers implicated in the underlying mechanisms of depression, schizophrenia, mania and certain neuropsychiatric diseases (11–13). The present study aimed to investigate the effects of fluvoxamine on the expression of these biomarkers by studying fluvoxamine-treated N2a cells and attempted to elucidate whether σ_1_ receptors mediate the pharmacological effects of fluvoxamine. Thus, BD1047, a σ_1_ receptor antagonist, was applied to fluvoxamine-treated N2a cells in order to observe its effects on the fluvoxamine-elicited pharmacological action.

## Materials and methods

### Reagents

Fluvoxamine and BD1047 were purchased from Tocris Bioscience (Minneapolis, MN, USA). Primary antibodies against mTOR, Camk2γ and GSK-3β were purchased from Cell Signalling Technology, Inc. (Danvers, MA, USA). The mouse N2a neuroblastoma cells were obtained from the Medical College, Soochow University (Suzhou, China).

### Cell culture

N2a cell culture was performed as described previously (14,15). The N2a cells were cultured in DMEM (Gibco, Grand Island, NY, USA) solution supplemented with 10% fetal bovine serum (FBS; Gibco), 0.3 mM L-glutamine and 50 U/ml penicillin/streptomycin. The N2a cells were randomly divided into three groups (six duplicates per group): DMEM group (D group), 0.5 μmol/l fluvoxamine group (F group) and 0.2 μmol/l BD1047 (σ_1_ receptor antagonist) + 0.5 μmol/l fluvoxamine group (BF group). Each culture well contained 2×10^5^ N2a cells. The N2a cells were prepared for analysis 48 h after the initiation of incubation,.

### Western blotting

The N2a cells were washed with phosphate-buffered saline (PBS). Protein levels were determined using the bicinchoninic acid method, according to the manufacturer’s instructions (Nanjing Kaiji Biochemistry Company, Nanjing, China). Briefly, bovine serum albumin (BSA) was applied as a standard protein. Prior to electrophoresis, a mixture of bromophenol blue and dithiothreitol (DTT; final concentration, 10 mM) was added to the samples. For western blotting, 50 μg of the total protein from each sample was separated by SDS-PAGE under reducing conditions. The proteins were then transferred onto polyvinylidene fluoride membranes (Nanjing Jiancheng Bioengineering Institute, Nanjing, China). The membranes were blocked for 2 h at room temperature using non-fat dried milk blotting-grade blocker and incubated overnight with primary antibodies. The primary antibodies used were goat anti-mTOR (1:1,000), rabbit anti-Camk2γ (1:1,000) and rabbit anti-GSK-3β (1:1,000). The primary antibodies were diluted in Tris-buffered saline (Thermo Fisher Scientific Inc., Rockford, IL, USA) containing 0.1% Tween-20 (TBS-T) and 2% BSA. Following extensive washing (three times for 15 min each in TBS-T), the mTOR, Camk2γ and GSK-3β protein levels were measured with horseradish peroxidase-conjugated rabbit anti-goat IgG (1:100,000 dilution) using enhanced chemiluminescence reagents (Beyotime, Nantong, China). Equal protein loading and transfer were assessed by subjecting each sample to western blotting for GAPDH with rabbit anti-GAPDH IgG (1:2,000 dilution).

### Statistical analysis

Data are expressed as the mean ± standard deviation and were analyzed using one-way analysis of variance, and post hoc analyses were performed using the least significant difference test. Statistical analysis was conducted using SPSS Software, version 17.0 (IBM, Chicago, IL, USA). P<0.05 was considered to indicate a statistically significant difference for all the data analyzed.

## Results

### Effects of fluvoxamine on the expression of mTOR, Camk2γ and GSK-3β in cultured N2a cells

**Figure 1:**
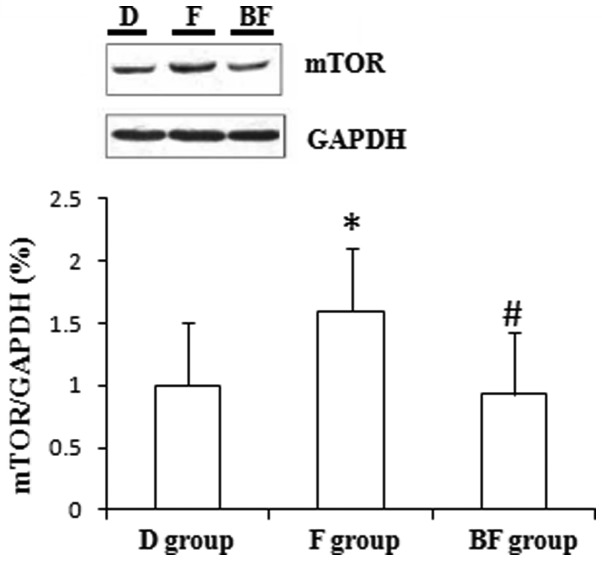
Expression of mTOR following fluvoxamine and/or BD1047 treatment in N2a cells. ^*^P<0.01 compared with D group; ^#^P<0.01 compared with F group. mTOR, mammalian target of rapamycin; D group, DMEM group; F group, fluvoxamine group; BF group, BD1047 + fluvoxamine group.

**Figure 2:**
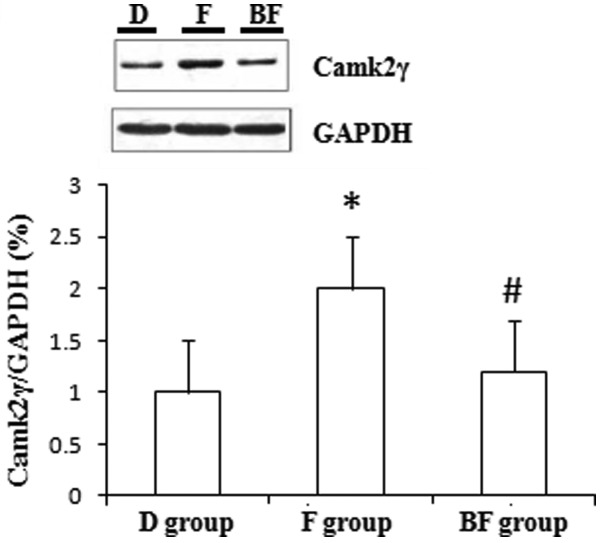
Expression of Camk2γ following fluvoxamine and/or BD1047 treatment in N2a cells. ^*^P<0.01 compared with D group; ^#^P<0.01 compared with F group. Camk2γ, Ca^2+^/calmodulin-dependent protein kinase 2γ; D group, DMEM group; F group, fluvoxamine group; BF group, BD1047 + fluvoxamine group.

**Figure 3:**
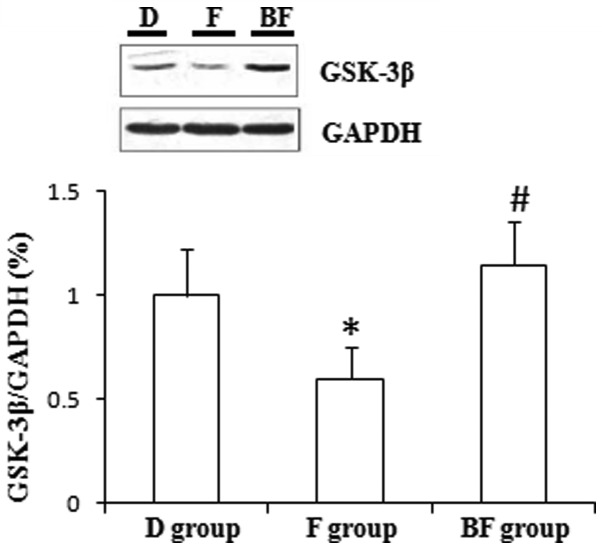
Expression of GSK-3β following fluvoxamine and/or BD1047 treatment in N2a cells. ^*^P<0.01 compared with D group; ^#^P<0.01 compared with F group. GSK-3β glycogen synthase kinase-3β; D group, DMEM group; F group, fluvoxamine group; BF group, BD1047 + fluvoxamine group.

The administration of fluvoxamine significantly increased the levels of mTOR and Camk2γ expression compared with those of the D group in the cultured N2a cells (P<0.01; Figs. 1 and 2). Fluvoxamine significantly decreased the levels of GSK-3β expression compared with those of the D group in the cultured N2a cells (P<0.01; Fig. 3).

### Effects of BD1047 on the fluvoxamine-elicited changes in the expression levels of mTOR, Camk2γ and GSK-3β in cultured N2a cells

The administration of BD1047 significantly decreased the levels of mTOR and Camk2γ expression compared with those of the F group in the cultured N2a cells (P<0.01; Figs. 1 and 2). Moreover, BD1047 significantly increased the levels of GSK-3β expression compared with those of the F group in the cultured N2a cells (P<0.01; Fig. 3).

## Discussion

Fluvoxamine is a widely used clinical antidepressant agent. Its primary pharmacological action is inhibition of the reuptake of serotonin, which ultimately increases the levels of serotonin in synaptic clefts and exerts therapeutic effects in patients with depression (1). N2a cells are a semi-adherent, fast growing, mouse neuroblastoma cell line. In the present study, N2a cells were used to investigate the pharmacological properties of fluvoxamine, and the results demonstrated that fluvoxamine significantly increased the mTOR and Camk2γ expression levels and decreased the GSK-3β expression levels.

It is generally acknowledged that fluvoxamine acts as an antidepressant agent with therapeutic effects that alleviate the symptoms of schizophrenia, obsession, bipolar depression and certain neuropsychiatric diseases (16–18). Increasing evidence has suggested that σ_1_ receptors may be pivotal in the mechanism of action of fluvoxamine in the treatment of schizophrenia and other psychiatric diseases (2). Notably, the results of the present study also demonstrated that BD1047, a σ_1_ receptor agonist, abolished the fluvoxamine-elicited pharmacological effects. This result was consistent with the expectations of the study.

mTOR is a type of protein that promotes the activity of neurons (11). The results of present study indicate that fluvoxamine increased the expression levels of mTOR in cultured N2a cells. mTOR stimulates the growth of neurons via increasing the expression levels of neurotropic factors and supplying nutrients (17). It has been demonstrated that upregulated mTOR expression levels in the prefrontal cortex are likely to be associated with the mechanisms of antidepressant effects, which facilitate the return of the depression-induced atrophic neurons to normal morphology and function (18). Additionally, a postmortem study has demonstrated downregulated mTOR expression levels in the brain tissues of depressed patients (19). Therefore, in the present study it was suggested that increased mTOR expression levels are probably involved in the mechanisms by which fluvoxamine exerts antidepressant effects. Furthermore, it was observed that BD1047 attenuated the Camk2γ-elicited increased in mTOR expression levels, which indicates that σ_1_ receptors are likely to be involved in the mechanism of action of fluvoxamine.

Camk2γ is a Ca^2+^-dependent protein kinase (13). However, there is little literature reporting whether its expression is associated with the mechanisms of psychiatric diseases. In the present study, it was observed that Camk2γ expression levels were significantly increased following treatment with fluvoxamine in cultured N2a cells. It is widely accepted that this antidepressant agent has neuroprotective effects. The results of the present study indicate that increased Camk2γ expression levels are probably associated with the neuroprotective and antidepressant effect of fluvoxamine. In addition, the results suggest that σ_1_ receptors are probably involved in the pharmacological effect of fluvoxamine on the expression of Camk2γ.

GSK-3β is serine/threonine kinase, which has been acknowledged as a pivotal target for the treatment of depression and mania (12). In the present study, it was observed that that fluvoxamine has the potential to inhibit GSK-3β and that σ_1_ receptors probably mediate this process, which suggests that fluvoxamine exerts its pharmacological effects via the serotonin pathway and also by stimulating σ_1_ receptors.

In conclusion, the results of this study demonstrate that fluvoxamine has an effect on the expression levels of mTOR, Camk2γ and GSK-3β, and that this process is likely to be associated with the activation of σ_1_ receptors. However, an *in vivo* study was not conducted to investigate whether σ_1_ receptor antagonists are able to attenuate the therapeutic effects of fluvoxamine. Future large-scale studies are required to elucidate the pharmacological properties of fluvoxamine.

## References

[1] Sugimoto Y, Tagawa N, Kobayashi Y, Mitsui-Saito K, Hotta Y, Yamada J (2012). Involvement of the sigma1 receptor in the antidepressant-like effects of fluvoxamine in the forced swimming test in comparison with the effects elicited by paroxetine. Eur J Pharmacol.

[2] Niitsu T, Iyo M, Hashimoto K (2012). Sigma-1 receptor agonists as therapeutic drugs for cognitive impairment in neuropsychiatric diseases. Curr Pharm Des.

[3] Hindmarch I, Hashimoto K (2010). Cognition and depression: the effects of fluvoxamine, a sigma-1 receptor agonist, reconsidered. Hum Psychopharmacol.

[4] Hayashi T, Su TP (2008). An update on the development of drugs for neuropsychiatric disorders: focusing on the sigma 1 receptor ligand. Expert Opin Ther Targets.

[5] Romieu P, Martin-Fardon R, Bowen WD, Maurice T (2003). Sigma 1 receptor-related neuroactive steroids modulate cocaine-induced reward. J Neurosci.

[6] Matsumoto RR, McCracken KA, Pouw B, Zhang Y, Bowen WD (2002). Involvement of sigma receptors in the behavioral effects of cocaine: evidence from novel ligands and antisense oligodeoxynucleotides. Neuropharmacology.

[7] Gebreselassie D, Bowen WD (2004). Sigma-2 receptors are specifically localized to lipid rafts in rat liver membranes. Eur J Pharmacol.

[8] Niitsu T, Fujisaki M, Shiina A (2012). A randomized, double-blind, placebo-controlled trial of fluvoxamine in patients with schizophrenia: a preliminary study. J Clin Psychopharmacol.

[9] Collina S, Gaggeri R, Marra A (2013). Sigma receptor modulators: a patent review. Expert Opin Ther Pat.

[10] Iyo M, Shirayama Y, Watanabe H (2008). Fluvoxamine as a sigma-1 receptor agonist improved cognitive impairments in a patient with schizophrenia. Prog Neuropsychopharmacol Biol Psychiatry.

[11] Kitagishi Y, Kobayashi M, Kikuta K, Matsuda S (2012). Roles of PI3K/AKT/GSK3/mTOR pathway in cell signaling of mental illnesses. Depress Res Treat.

[12] Li X, Jope RS (2010). Is glycogen synthase kinase-3 a central modulator in mood regulation?. Neuropsychopharmacology.

[13] Tombes RM, Faison MO, Turbeville JM (2003). Organization and evolution of multifunctional Ca^2+^/CaM-dependent protein kinase genes. Gene.

[14] Strenge K, Schauer R, Kelm S (1999). Binding partners for the myelin-associated glycoprotein of N2A neuroblastoma cells. FEBS Lett.

[15] Björkdahl C, Sjögren MJ, Winblad B, Pei JJ (2005). Zinc induces neurofilament phosphorylation independent of p70 S6 kinase in N2a cells. Neuroreport.

[16] Apter A, Ratzoni G, King RA (1994). Fluvoxamine open-label treatment of adolescent inpatients with obsessive-compulsive disorder or depression. J Am Acad Child Adolesc Psychiatry.

[17] Chen A, Xiong LJ, Tong Y, Mao M (2013). Neuroprotective effect of brain-derived neurotrophic factor mediated by autophagy through the PI3K/Akt/mTOR pathway. Mol Med Rep.

[18] Li N, Lee B, Liu RJ (2010). mTOR-dependent synapse formation underlies the rapid antidepressant effects of NMDA antagonists. Science.

[19] Jernigan CS, Goswami DB, Austin MC (2011). The mTOR signaling pathway in the prefrontal cortex is compromised in major depressive disorder. Prog Neuropsychopharmacol Biol Psychiatry.

